# The Influence of B Cell Depletion Therapy on Naturally Acquired Immunity to *Streptococcus pneumoniae*

**DOI:** 10.3389/fimmu.2020.611661

**Published:** 2021-01-28

**Authors:** Giuseppe Ercoli, Elisa Ramos-Sevillano, Rie Nakajima, Rafael Ramiro de Assis, Algis Jasinskas, David Goldblatt, Philip Felgner, Gisbert Weckbecker, Jeremy Brown

**Affiliations:** ^1^ Centre for Inflammation and Tissue Repair, UCL Respiratory, Division of Medicine, University College Medical School, Rayne Institute, London, United Kingdom; ^2^ Vaccine Research and Development Center, Department of Physiology and Biophysics, University of California Irvine, Irvine, CA, United States; ^3^ Department of Immunobiology, UCL Great Ormond Street Institute of Child Health, NIHR Biomedical Research Centre, London, United Kingdom; ^4^ Novartis Institute for BioMedical Research, Novartis, Basel, Switzerland

**Keywords:** *Streptococcus pneumoniae*, anti-protein antibody, colonization, B cell depletion, CD20

## Abstract

The anti-CD20 antibody Rituximab to deplete CD20+ B cells is an effective treatment for rheumatoid arthritis and B cell malignancies, but is associated with an increased incidence of respiratory infections. Using mouse models we have investigated the consequences of B cell depletion on natural and acquired humoral immunity to *Streptococcus pneumoniae*. B cell depletion of naïve C57Bl/6 mice reduced natural IgM recognition of *S. pneumoniae*, but did not increase susceptibility to *S. pneumoniae* pneumonia. ELISA and flow cytometry assays demonstrated significantly reduced IgG and IgM recognition of *S. pneumoniae* in sera from mice treated with B cell depletion prior to *S. pneumoniae* nasopharyngeal colonization compared to untreated mice. Colonization induced antibody responses to protein rather than capsular antigen, and when measured using a protein array B cell depletion prior to colonization reduced serum levels of IgG to several protein antigens. However, B cell depleted *S. pneumoniae* colonized mice were still partially protected against both lung infection and septicemia when challenged with *S. pneumoniae* after reconstitution of their B cells. These data indicate that although B cell depletion markedly impairs antibody recognition of *S. pneumoniae* in colonized mice, some protective immunity is maintained, perhaps mediated by cellular immunity.

## Introduction

B cells (of B lymphocytes) are a major component of the adaptive immunity, and are essential for the secretion of antibodies directed against “non-self” antigens. There are multiple B cells subtypes that can also be involved in antigen presentation (APC), the secondary immune response (memory B cells), and suppression of immune responses (Bregs) (1–3). Dysregulation of B cell mediated immunity and the inappropriate production of autoimmune antibodies can result in autoimmune diseases, and there are several B cell neoplastic conditions including lymphoma, chronic lymphocytic leukaemia and myeloma (4, 5). As a consequence, antibody-mediated B cell depletion therapies have been developed as effective treatments for multiple autoimmune diseases and B cell malignancies with an increasing range of clinical applications (6). B cell depletion therapy involves the parenteral administration of monoclonal antibodies that bind receptors expressed on the surface of the B cells to induce cell death. Depending on the treatment strategy, the therapy can target a high proportion of the B cell population (e.g. anti-CD20) (7) or specific subsets (e.g. antiCD19, anti-CD22) (8, 9). B cell depletion therapies are used to treat rheumatoid arthritis, systemic lupus erythematosus, multiple sclerosis, immune thrombocytopenia, non-Hodgkin’s lymphoma or chronic lymphocytic leukaemia (10, 11). However, a common complication of autoimmune and malignant disease are serious respiratory infections, which could be exacerbated by depleting the B cell repertoire by reducing levels of protective antibodies (12). Indeed, B cell depletion is associated with an increased incidence of respiratory tract infections that can lead to bronchiectasis (13–15). Despite the increased risk of respiratory infections, there are few data on how B cell therapy affects the subsets of specific antibodies to respiratory pathogens.

B cell depletion does not eliminate completely B cell immunity. Long live plasma cells (CD20-) and circulating antibodies appear to be maintained following Rituximab (RTX) treatment, and although the numbers of CD20+ memory B cells are reduced, they are not completely abrogated (16) and upon restimulaton they can rapidly expand and differentiate into antibody- producing cells. The main determining factor seems to be duration of B cell depletion therapy and for how long B cell lymphopenia is sustained, with possible impact on Th17 response and regulatory T cells (17, 18). Vaccination is the main strategy in place to boost immunity against pathogens and prevent infections in RTX treated patients. The timing of vaccination seems to be important, with evidence showing that following rituximab treatment vaccine responses are impaired for up to 6 months (19, 20). Hence, guidelines recommend vaccination against major pathogens 2–3 weeks before planned B cell depletion (21).

A major respiratory pathogen is *Streptococcus pneumoniae*, the dominant bacterial pathogen causing pneumonia, which is also a common cause of septicemia and meningitis (22). B cell dependent immunity to *S. pneumoniae* is mediated through several mechanisms. The B1a B cell subset produces natural IgM antibodies that are largely thought to target cell wall phosphocholine and improve complement-mediated systemic immunity against *S. pneumoniae* (23). Asymptomatic nasopharyngeal colonization with *S. pneumoniae* can induce antibody to both protein and/or capsular antigens (24–27). Recent data suggests anti-protein antibody probably forms the dominant component of naturally acquired IgG adaptive immunity against *S. pneumoniae* in humans (24, 28, 29), and have identified the range of antigens recognized in normal human sera (24, 30, 31). Despite the clinical importance of respiratory pathogens especially in immunosuppressed subjects, at present, there are limited data on the consequences of the different modalities of B cell depletion on antibody-mediated immunity to *S. pneumoniae.* In this study, we have developed a mouse model of B cell depletion and tested the consequences of low levels of B cells on natural IgM and the development of colonization induced antibody mediated immunity to *S. pneumoniae* to subsequent pneumonia challenge.

## Materials and Methods

### Bacterial Strains

*Streptococcus pneumoniae* strains D39, BHN418 6B, and TIGR4 were used for this study (capsular serotypes 2, 6B, and 4, respectively). All pneumococcal strains were cultured in Tryptic Soy Broth (TSB, Becton Dickinson) or on blood agar plates consisting of Columbia Agar (Becton Dickinson) supplemented with 3% v/v defibrinated horse blood at 37°C in 5% CO_2_.

### Animal Models

Five-week-old, female, inbred C57Bl/6 mice from Charles River (Margate, Kent CT9 4LT UK) were used in this study. Before use, mice were housed for at least 1 week under standard conditions, in the Biological Service animal facility at the University College of London, according to its guidelines for the maintenance of laboratory animals. No randomization or blinding was performed. All animal procedures were approved by the local ethical review process and conducted in accordance with the relevant, UK Home Office approved, project license (PPL70/6510). For the colonization model, mice were anaesthetized using isoflurane and then inoculated intranasally using a dose of 1 x 10^7^ CFU in 10 µl volume (25, 32). For the pneumonia with secondary septicemia model, mice were anaesthetized using isoflurane and then infected intranasally using a dose of 1 x 10^7^ CFU in 50 µl volume (25, 32). Mice were culled 24 h after infection. Mouse organs were homogenized in 1 ml of PBS for quantification of colony forming units (CFU) and flow cytometry analysis. Blood samples from mice were collected by tail bleeds or cardiac puncture under terminal anaesthesia, and treated with 100 U/ml of heparin (Sigma Aldrich, UK) to prevent blood coagulation.

### B Cell Depletion Treatment and Flow Cytometry Analysis of Splenocytes

B cell depletion on mice was performed by IV or IP injection of aCD20 antibody (Rat IgG2b, κ, SA271G2, BioLegend) (33). Different doses were used depending on the route of injection (50–100 µg for IV injection and 25–100 µg for IP injections). Isotype control rat IgG was used as negative control. The effects of B cell depletion treatment was analyzed using flow cytometry on splenocytes. Splenocytes were prepared by passing mouse spleens through a cell strainer to obtain single cell suspensions; red blood cells were removed using a red blood cell lysis buffer (RBC). Splenocytes were stained using fluorescently conjugated antibodies to define the different immune cell populations using the following surface markers: CD19 (B cells, BioLegend, 115529), CD3 (T cells, BioLegend, 100219), Ly-6G (neutrophils, BioLegend, 127615), CD11c (monocytes, BioLegend, 117317), and the B cell subset markers CD23, CD21 (BioLegend, 123415), CD5 (ThermoFisher, 11-0051-81), and IgM (BioLegend, 406525). Samples have been analyzed using a BD FACSVerse and data have been processed using FlowJo software for Windows (version 10).

### Whole Cell Elisa and Flow Cytometry IgG and IgM Binding Assays

Antibody recognition of *S. pneumoniae* was assessed using previously described whole cell ELISAs and flow cytometry assays (32). Briefly, for whole cell ELISAs *S. pneumoniae* were grown to an OD_600_ of approximately 0.4–0.8, washed and resuspended to an OD_600_ of 0.4 in PBS, 50 μl/well were added to microtiter plates and incubated overnight at RT before fixation in 4% formaldehyde for 10 min. Plates were washed and incubated with a 1:100 dilution of murine antiserum for 1 h at 37°C and using HRP-conjugated goat anti-mouse IgG (abCam, ab6789) for detection. For flow cytometry antibody binding assays live *S. pneumoniae* (1 x 10^6^ CFU) were incubated for 30 min at 37°C with 10% mouse serum. Fluorescently labelled anti-mouse IgG (BioLegend, 405308) and IgM (BioLegend, 406505) were used to detect antibody binding to the bacterial surface using a BD FACSVerse instrument.

### Measurement of IgG Recognition of Specific S. pneumoniae Protein Antigens

Antibody levels to selected *S. pneumoniae* protein antigens (5 μg/ml) were measured using a multiplexed electroluminescence assay as previously described (24, 34) using a Meso Scale Discovery (MSD; MD, USA) platform assay. After incubation of each antigen-coated plate with blocking agent, washing, and incubation with diluted test serum for 45 min at room temperature, the plates were washed and MSD assay sulfo tag-labeled goat anti-mouse IgG secondary antibody (MSD, R32AC-1) was added for reading using an MSD Sector Imager 2400 or 6000 apparatus.

A *S. pneumoniae* protein array was constructed containing 289 proteins. Proteins were selected based on having a high level of conservation in a panel of >600 *S. pneumoniae* strains (31), and included the majority of the conserved proteins identified by Croucher et al. that were recognized to significant levels by IgG present in human sera obtained from healthy adults (31). The list of selected proteins is reported in the **Supplementary Materials File**. The array was constructed using genes amplified from genomic DNA (strain TIGR4) and cloned into a T7 expression vector. Proteins were expressed by incubating the plasmids for 16h in *E. coli*-based *in vitro* transcription/translation (IVTT) reactions (RTS E. coli HY 100 kit, biotechrabbit GmbH, Germany). Proteins were tested for expression by Western blotting using antibodies against N-terminal poly-histidine (His) after printing onto nitrocellulose coated glass AVID slides (Grace Bio-Labs), using an Omni Grid 100 microarray printer (Genomic Solutions). Arrays were probed with mouse serum samples diluted 1:100 in protein array blocking buffer (GVS, Sanford, ME) and supplemented with *E. coli* lysate. Images were acquired and analyzed using an ArrayCAM^®^ Imaging System from Grace Bio-Labs. Signals were quantified using QuantArray software utilizing automatic local background subtraction for each spot. ‘‘No DNA’’ controls consisting of *E. coli* IVTT reactions without addition of DNA were averaged and used to subtract background *E. coli* reactivity. All results presented are expressed as Median Fluorescence Intensity (MFI) (35, 36).

### Statistical Analysis

Statistical analyses were conducted using Prism 7 (Graph Pad, USA). Unless otherwise stated, data are presented as means, and error bars represent standard deviations. Parametric data were analyzed using Student’s T test or ANOVA, and nonparametric data using the Mann-Whitney U test. For CFU analysis on infected mice, Fisher test was also applied to compare numbers of infected versus uninfected animals.

## Results

### B Cell Depletion in a Mouse Model

**Figure 1:**
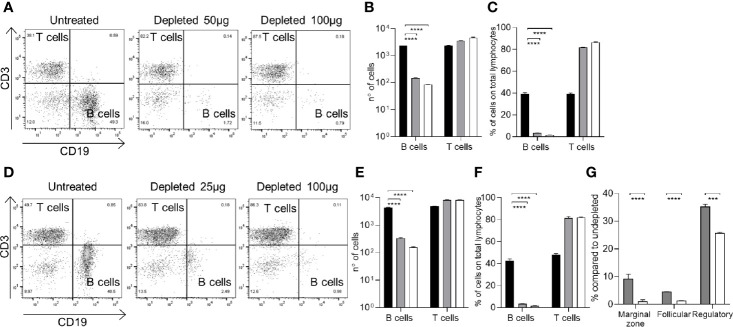
Effects of IP or IV administration of aCD20 on splenocyte B cell populations in C57Bl/6 mice. Splenocyte B cell (anti-CD19 antibody) and T cell (anti-CD3 antibody) populations were quantified using flow cytometry 7 days after the administration of the aCD20 antibody (SA271G2 BioLegend). **(A–C)** IV administration of either 50µg or 100µg aCD20, showing representative flow cytometry plots **(A),** total cell numbers **(B)**, and cell population proportions **(C)**. **(D, E)** IP administration of either 25 µg or 100 µg aCD20, showing representative flow cytometry plots **(D)**, total cell numbers **(E),** and cell population proportions **(F)**. **(G)** Effects of B cell depletion on B cell subsets for IP treated mice; marginal zone (CD5-CD21+CD23-IgM+), follicular (CD5-CD23+IgM-) and regulatory (CD5+CD21+IgM+) B cell populations are shown. For all histograms columns represent means, error bars SDs: black bars, undepleted; gray bars, low-dose depleted; white, high-dose depleted (n=3). Mann Whitney U test was used for statistical analysis (***P < 0.001, ****P < 0.0001).

**Figure 2:**
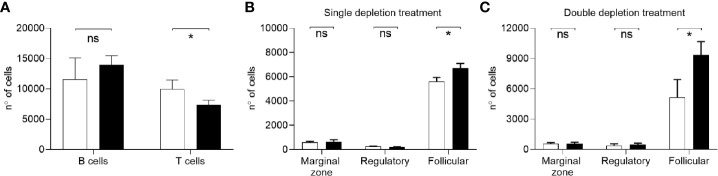
Reconstitution of B cell population after depletion. **(A)** Flow cytometry analysis of total number of B (CD19+) and T cells (CD3+) in single cell spleen preparations from C57Bl/6 mice colonized with *S. pneumoniae* 3 days after B cell depletion treatment. B cell reconstitution has been assessed 26 days after aCD20 antibody IP administration (3 mice/group). Undepleted mice are shown in black, depleted in white. **(B, C)** Reconstituted B cell subset populations in splenocytes after one **(B)** or two **(C)** depletion treatments were analyzed by flow cytometry 31 and 26 days after depletion, respectively. Marginal zone (CD5-CD21+CD23-IgM+), follicular (CD5-CD23+IgM-), and regulatory (CD5+CD21+IgM+) B cells populations are shown. Depleted/vaccinated (white bars), undepleted/vaccinated (black bars) mice were analyzed (n=6/group). Bars represent mean values for each group, error bars indicate SDs and Mann Whitney U test was used for statistical analysis (*P < 0.05, ns, not significant).

To mimic the effect of B cell depletion therapy, mice were injected with a mouse anti-CD20 Ab. C57Bl/6 mice were injected with a single dose of anti-CD20 antibody (Rat IgG2b, κ, SA271G2, BioLegend) using intravenous (IV) and intraperitoneal (IP) routes of administration and two different doses for both conditions. IV injection of C57Bl/6 mice was used to mimic clinical route of administration of Rituximab in patients and compared to the IP route, which could provide an easier and more reproducible animal model of B cell depletion. Splenic lymphocyte repertoires were analyzed using flow cytometry 7 days after treatment. IV injection of 50 µg or 100 µg of anti-CD20 antibody depleted 92% and 96% of total B cells, respectively (**Figures 1A–C**). IP injection of 25 µg or 100 µg of anti-CD20 depleted 93% and 98% of total B cells, respectively (**Figures 1D–F**). The effect of IP anti-CD20 on B cell subsets was also investigated and demonstrated relative preservation of Bregs (CD5+CD21+IgM+) (37, 38) but major reductions in marginal zone (CD5-CD21+CD23-IgM+) (38–40) and follicular (CD5-CD23+IgM-) (38, 39) B cell numbers (**Figure 1G**). Splenocytes from mice colonized with *S. pneumoniae* following IP administration of anti-CD20 antibody were analyzed 26 days after depletion and showed almost complete reconstitution of the B cell population (**Figure 2A**). The reconstituted splenic B cell subset populations were further analyzed after IP administration of one or two doses (10 days apart) of anti-CD20 (**Figures 2B, C** **)**. Although there were a similar number of splenic B cells compared to controls, reconstitution of the follicular B cell population was slightly impaired in mice 26 days after they had been given two doses of anti-CD20. Overall, these data show that either IV or IP administration of anti-CD20 caused very significant reductions in the numbers of splenic B cells, which was largely reversed by 21 days after antibody treatment.

### B Cell Depletion Impairs Natural IgM Recognition of *S. pneumoniae*

**Figure 3:**
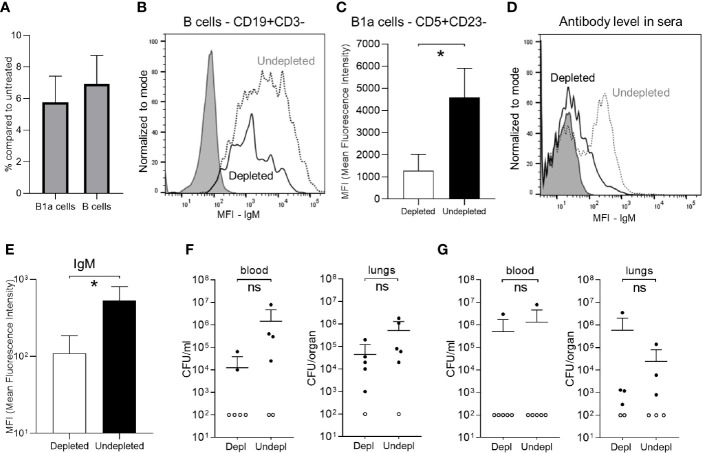
Effect of B cell depletion on natural IgM mediated immunity against *S. pneumoniae.* Flow cytometry analysis of splenocytes recovered from C57B/6 mice 7 days after IP administration of 100ug of aCD20 antibody (six mice/group). **(A)** Percentage of persisting natural IgM secreting cells (B1a, CD5+CD23-) and total B cells (CD19+CD3-) compared to undepleted controls. **(B)** A representative flow cytometry plot of the relative level of IgM expression in splenic B cells from aCD20 treated (solid line) and untreated controls (dotted line). Unstained sample data are also shown (gray filling). **(C)** IgM expression level (Mean fluorescence intensity - MFI) on B1a cells (CD5+CD23-) in the depleted (white bar) versus undepleted mice (black bar). **(D)** A representative flow cytometry plot of IgM binding to *S. pneumoniae* 6B strain incubated in serum from undepleted (dotted line) and B cell depleted (solid line) mice; unstained samples (gray filling). **(E)** Mean MFI of IgM binding to *S. pneumoniae* 6B strain in serum from B cell depleted (white column) or undepleted (black columns) C57B/6 mice. **(F, G)** Effects of B cell depletion of immune naïve C57B/6 mice on susceptibility to *S. pneumoniae* pneumonia. Mice were inoculated intranasally with 2 x 10^6^ CFU *S. pneumoniae* 6B **(F)** and D39 strain **(G)** CFU in 50 ul PBS under deep anaesthesia and CFU were measured at 24 h by plating serial dilutions of blood and lungs. For all graphs, bars represent the mean values for each group, error bars indicate standard deviations and Mann Whitney U test was used for statistical analysis (*P < 0.05, ns, not significant).

To assess the effects of B cell depletion on natural IgM responses to *S. pneumoniae*, C57B/6 mice (a strain known to have significant levels of natural IgM against *S. pneumoniae*) (23) without prior exposure to *S. pneumoniae* were treated with anti-CD20. The natural IgM secreting B cells subset was analyzed in isolated splenocytes. As expected the number of B cells (CD19+CD3-) dropped significantly in the depleted group (**Figure 3A**). Flow cytometry also demonstrated a 92-94% reduction in the B1a subset (natural IgM secreting cells, CD5+CD23-) (38, 41), a similar reduction to the effects on the whole B cell population (**Figure 3A**). B cell depletion also reduced the level of IgM expression by the total B cell population (**Figure 3B**) and for the B1a cells subpopulation (**Figure 3C**). The quantity of IgM binding to the *S. pneumoniae* 6B strain was reduced when bacteria were incubated in serum from B cell depleted mice (**Figures 3D, E**), indicating B cell depletion resulted in impaired recognition of *S. pneumoniae* by natural IgM. However, when challenged 7 days after with a sublethal dose by intranasal inoculation of the 6B or the D39 *S. pneumoniae* strain (2 x 10^6^ Colony Forming Units - CFU), B cell depleted naïve C57Bl/6 mice did not have increased susceptibility to *S. pneumoniae* pneumonia (**Figures 3F, G**).

### B Cell Depletion Prior to Colonization Reduces Antibody Responses to *S. pneumoniae*

**Figure 4:**
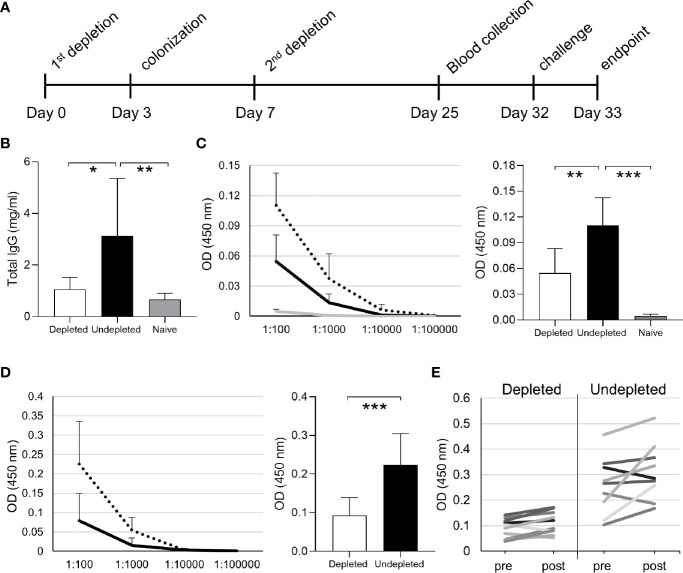
Antibody response in depleted/colonized mice. **(A)** The full timeline of B cell depletion treatment with aCD20 antibody followed by intranasal pneumococcal colonization is reported. **(B)** Total IgG levels of the depleted colonized and not depleted colonized groups. IgG specific to 6B **(C)** and TIGR4 **(D)** pneumococcal strains measured by whole cell ELISA assay are reported both for serially diluted sera and at 1:100 dilution. Depleted (solid line) and undepleted (dotted line) mice IgG levels are shown for both strains, uncolonized control IgG levels (gray line) is only shown for 6B. **(E)** Comparison of anti-pneumococcus IgG levels of individual mouse sera before and after challenge are also reported. Bars represent the average values for each group. Error bars indicate standard deviations and Mann Whitney U test was used for statistical analysis (*P < 0.05, **P < 0.01, ***P < 0.001).

**Figure 5:**
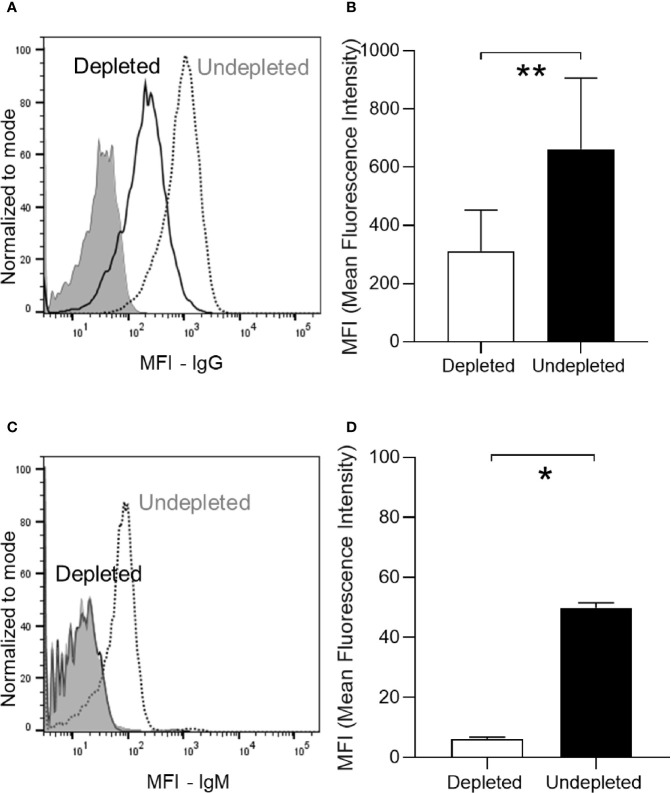
Antibody response in depleted/colonized mice. **(A)** Anti-pneumococcus (6B strain) specific IgG level has been measured by serum deposition assay in individual mice as shown by a representative FACS histogram plot. The dotted line represents the undepleted/colonized, the solid line the depleted/colonized and gray filled line the non-colonized control sample. **(B)** The average MFI for each group are also reported in panel **(B)**. **(C, D)** Anti-pneumococcus (6B strain) IgM have been also measured by serum deposition assay on pooled sera of each group. Bars represent the average values for each group. Error bars indicate standard deviations and Mann Whitney U test has been used for statistical analysis (*P < 0.05, **P < 0.01).

The effects of B cell depletion on colonization-induced immunity was assessed in C57Bl/6 mice using the schedule shown in **Figure 4A**. Mice were B cell depleted by IP injection of 50 µg anti-CD20 followed after 3 days by a single colonization event with 5 x 10^6^ CFU of the *S. pneumoniae* 6B strain and an additional B cell depletion treatment (IP 50 µg) after a further 4 days. The mice were challenged using the homologous *S. pneumoniae* 6B strain pneumonia model 25 days after the second B cell depletion treatment. Serum was obtained 18 days after the second B cell depletion treatment and at the end point of the experiment 1 day post-challenge. Total IgG levels in B cell depleted colonized mice were similar to uncolonized controls, showing that B cell depletion did not affect the overall quantity of IgG after recovery of the B cell population (**Figure 4B**). However, B cell depleted mice lost the increase in total IgG observed for colonized mice not previously treated with B cell depletion. When assessed using whole cell ELISAs there was reduced recognition of the homologous *S. pneumoniae* strain 6B and the heterologous TIGR4 strain (**Figures 4C, D**) in serum obtained from mice after B cell depletion compared to colonized undepleted controls. The boosting effect of the bacterial challenge on the IgG level was also analyzed by comparing ELISA data for sera collected pre- and post-pneumoniae challenge with the 6B strain. Overall, there was only a slight increase in IgG recognition of *S. pneumoniae* in post- compared to pre-challenge sera for both depleted and undepleted mice (**Figure 4E**). The effect of B cell depletion on *S. pneumoniae* opsonization with IgG and IgM was assessed using a flow cytometry assay. This demonstrated that B cell depletion treatment reduced the amount of both IgG and IgM binding when the *S. pneumoniae* 6B strain were incubated in post-colonization sera (**Figures 5A–D**). IgG binding of the same set of sera was also tested against the TIGR4 strain, and showed a significant reduction in the B cell depleted group (average MFI undepleted 184.5 ± 113.9, depleted 70.8 ± 27.22).

### B Cell Depletion Reduced Anti-Protein Antigen Antibody Responses Induced by *S. pneumoniae* Colonization

**Figure 6:**
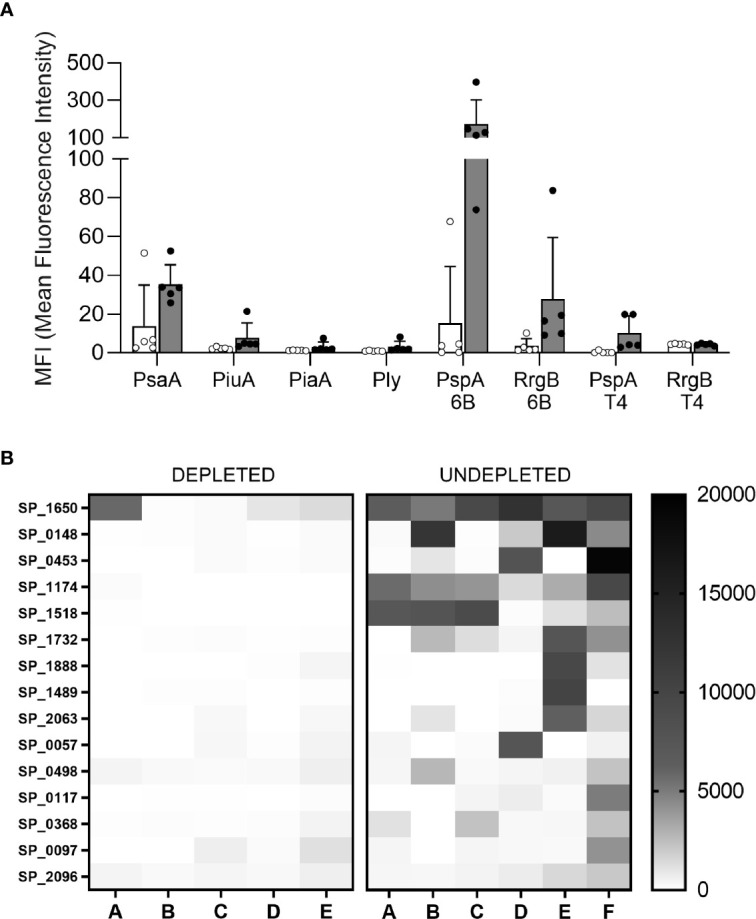
Antigen specific response. **(A)** Meso Scale Discovery (MSD) has been used to measure the specific IgG response to 6 pneumococcal antigens in the sera of depleted (white) and not depleted (gray filled) C57Bl/6 mice colonized with *S. pneumoniae* 6B strain. Error bars indicate standard deviations, each point represents individual mice. **(B)** A protein array containing 289 conserved pneumococcal antigens has been also probed with the individual mice sera, the IgG level against the top 15 antigens is showed for each mouse of the depleted (left) and undepleted (right) groups. Level of recognition measured in median fluorescence intensity goes from minimum = 0 (white) to maximum = 20000 (black) and it is shown in boxes that represent the average values of each single antigen for individual mice.

The effects of cell depletion on colonization induced antibodies to specific *S. pneumoniae* antigens was assessed using Meso Scale Discovery (MSD) multiplex assays for anti-capsular antibody levels and for anti-protein responses to a panel of known *S. pneumoniae* surface exposed immunogenic proteins. There were no detectable antibody responses to serotype 6B capsular antigen in any mice (data not shown). When measured using MSD, in B cell depleted mice colonization-induced there were substantial decreases in the levels of specific IgG for the protein antigens PsaA, PiuA, PspA, and RrgA compared to the undepleted colonized control group (**Figure 6A**). The effects of B cell depletion on IgG responses to *S. pneumoniae* protein antigens was explored further using a protein array containing 289 pneumococcal antigens (**Supplementary Materials**), including the majority of antigens previously shown to be recognized by IgG in normal human sera (24, 30, 31). Comparison of the IgG-specific signal for each antigen after incubation with sera from the B cell depleted mice demonstrated a statistically significant reduction in mean recognition of the top 15 recognized antigens (**Figure 6B**, **Table 1**). In particular, significant reductions have been observed in antigen-specific IgG recognizing the protein antigens PsaA, Sp_0148, Sp_1174, MltG, and StkP compared to undepleted controls (**Figure 6B**, **Table 1**).

**Table 1:** IgG levels against pneumococcal antigens measured by protein array.

Antigen annotation	Gene name	Gene function	Median fluorescence binding (Average ± SD)		P value
			Undepleted	B cell depleted	
SP_1650	*psaA*	Manganese ABC transporter substrate-binding lipoprotein	8331,8 ± 2660,3	2366 ± 3549,1	0.011**
SP_0148		ABC transporter, substrate-binding protein	5790,6 ± 6701	108,2 ± 95,3	0.034*
SP_0453		ATP-binding cassette (Glutamine)	4704,1 ± 7706,9	98 ± 100,9	0.218
SP_1174		Unknown	4627,9 ± 2739,4	31,6 ± 70,7	0.005**
SP_1518	*mltG*	Endolytic murein transglycosylase	4595,9 ± 3804	0,6 ± 1,34	0.025*
SP_1732	*stkP*	Serine/threonine-protein kinase	2650,9 ± 2831	56,4 ± 45,9	0.038*
SP_1888	*amiE*	oligopeptide ABC transporter	1745,2 ± 3769,8	108,5 ± 203,8	0.362
SP_1489	*tuf*	Elongation factor Tu	1678,4 ± 4057	32 ± 29,7	0.392
SP_2063		LysM domain protein	1445,5 ± 2333,2	132,6 ± 181,9	0.245
SP_0057	*strH*	endo-beta-N-acetylglucosaminidase	1419,5 ± 3005,7	198,2 ± 248,8	0.392
SP_0498		Putative endo-beta-N-acetylglucosaminidase	1062,8 ± 1111,8	398,2 ± 222,1	0.090
SP_0117	*pspA*	Inhibition of C3 deposition	1029,3 ± 1934,1	35,4 ± 59,8	0.284
SP_0368		Endo-alpha-N-acetylgalactosaminidase	1026,7 ± 1036,4	181,6 ± 215,5	0.109
SP_0097		Conserved domain protein	875,7 ± 1576,4	490,2 ± 628,4	0.622
SP_2096		N-acetyldiaminopimelate deacetylase	872,8 ± 718,6	441,6 ± 182,6	0.227
Average for top 15 antigens			2790,47 ± 3065,7	311,9 ± 389,1	0.0023**

Level of IgG recognition of specific S. pneumoniae protein antigens in sera obtained 30 days after colonization of C57B/6 mice with the 6B strain with or without prior B cell depletion. Data are presented for the top 15 antigens recognized in undepleted sera, and for the mean overall strength of antigen recognition. Data are presented as median fluorescence intensity and were compared using unpaired t-test (*P < 0.05, **P < 0.01). The whole dataset containing all the 289 antigens is reported in the **Supplementary Material File**.

### B Cell Depletion Only Partially Impaired Colonization Induced Protection Against *S. pneumoniae* Pneumonia

**Figure 7:**
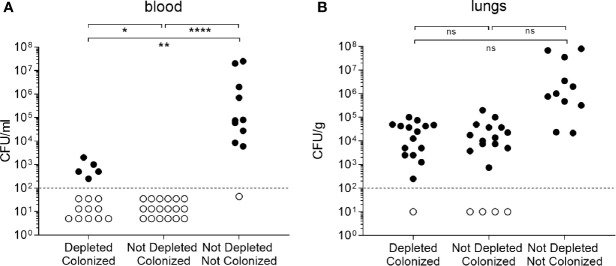
B cell depletion effect on colonization. **(A)** CFU measured from blood of C57Bl/6 mice 24 h after intranasal infection with 6B strain. Mice were divided in three groups depending on the pre-infection treatment received: depleted colonized (n=16), not depleted colonized (n=18) and not depleted not colonized (n=11). At day 32, pneumococcal pneumonia was induced infecting intranasally the mice with 6B strain. **(B)** CFU measured from the same experiment in lungs homogenates. Error bars indicate standard deviations and Fisher’s exact test was used for statistical analysis (*P < 0.05, **P < 0.01, ****P < 0.0001).

Colonized mice were challenged by intranasal administration with the *S. pneumoniae* 6B strain. At 24 h post-challenge all uncolonized mice had developed septicemia, and no CFU were found in the blood of the colonized B cell undepleted control mice, whereas 5/16 (31.3%) B cell depleted colonized mice developed systemic infection. Both B cell depleted and undepleted colonized mice had an approximately 1 log_10_ reduction in lung CFU compared to uncolonized controls (**Figure 7**). These data suggest B cell depletion before colonization partially impairs colonization-induced protective adaptive immunity against septicemia, but had no effect on colonization-induced immunity to lung infection.

## Discussion

In this study, a mouse model was developed to study the effect of B cell depletion therapy on the immunity to *S. pneumoniae*. The administration of anti-CD20 antibody in mice induced almost complete depletion of the B cell repertoire mimicking what happens in patients undergoing comparable treatments. A small proportion of the B cells were preserved after treatment; in particular, over 30% of the Bregs subset were maintained, a similar result to previous data obtained using a mouse model (42). Bregs have immunosuppressive properties, they mainly produce IL-10 contributing to the anti-inflammatory activity and their expansion have been associated with the persistence of bacterial infections (43). Other B cell populations escaping the depletion treatment are progenitor B cells and antibody-secreting cells (plasmablasts and plasma cells) which do not express the CD20 marker. Persistence of plasma cells with a lifespan of 6 months after anti-CD20 antibody could provide a degree of continued immunity through secretion of antigen-specific IgG and IgA (44). We also analyzed the reconstitution of the B cell repertoire, showing full reconstitution of marginal zone and regulatory B cell subsets within 4 weeks of B cell depletion treatment, while follicular B cells failed to return to untreated levels. Marginal zone B cells activate early IgM response against *S. pneumoniae* and by interacting with SIGN-R1 macrophages promote bacterial clearance (45). After a single depletion treatment, we observed 83% of follicular B cells reconstituted, while after 2 subsequent depletion treatments, only 55% of the follicular B cells were observed compared to untreated mice. Although complete reconstitution of the follicular B cell subsets could occur after a longer period post-B cell depletion treatment, our data indicate that anti-CD20 administration has the most marked effect on mature B cells in the follicles with potential consequences for T cell dependent antibody responses (46–48).

Both innate and adaptive immunity to *S. pneumoniae* were studied using the depleted mouse model. Natural IgM, known to be an important component of innate immunity to *S. pneumoniae* (23, 49), were analyzed using mice with no prior exposure to *S. pneumoniae*. We observed B cell depletion reduced the number of natural IgM secreting cells (B1a) and impaired opsonization of *S. pneumoniae* with IgM in serum, but had no effect on susceptibility to infection challenging the mice with a sublethal dose of pneumococci. These data indicate that the reduction in natural IgM secreting B cells post-B cell depletion did not result in a discernible weakening of innate immunity to *S. pneumoniae*. Multiple other aspects of innate immunity provide protection against *S. pneumoniae* (e.g. other complement activators, neutrophils, and macrophage subpopulations of the spleen and liver) (23, 50–52) and it is likely this redundancy prevented the identification of any effect of depletion of B cells.

Naturally acquired adaptive immunity to *S. pneumoniae* is mediated by antibodies to the polysaccharide capsule and/or multiple protein antigens, and by Th17 and Th1 cellular immunity (25, 28). In this study, in line with previous work (24), we observed that anti-capsule response in the mouse model of colonization was extremely low, but antibody responses were induced to multiple protein antigens. B cell depletion had a profound effect on antibody recognition of *S. pneumoniae* after colonization, including the heterologous TIGR4 strain further suggesting recognition was dependent on anti-protein rather than anti-capsular responses. Which protein antigen antibody responses were impaired by B cell depletion was analyzed in detail using an MSD assay and a protein array that contains 289 conserved *S. pneumoniae* protein antigens including those that are recognized in human sera. In the colonized non-B cell depleted mouse sera, some antigens were recognized by serum IgG obtained from all individual mice (e.g. Sp_1650 and Sp_1174), whereas IgG responses to other antigens were much more variable between mice (e.g. Sp_0148 and Sp_0453). B cell depletion decreased levels of specific anti-protein IgG to all antigens detected in untreated colonized mice, although the strength of the effect varied between antigens from approximately 50%–95% reductions in median fluorescence intensity. The mouse model we have used does not replicate the normal human situation, in which previous childhood colonization with *S. pneumoniae* results in significant humoral and T cell mediated immunity. However, recolonization events as an adult boost these pre-existing adaptive immune responses (53) which is likely to be important for maintenance of immunity to *S. pneumoniae*. Our data suggest that B cell depletion in humans around the time of a re-colonization event is likely have a marked negative effect on the boosting of humoral responses to *S. pneumoniae* protein antigens, and consequently could weaken protection against future infection. Future experiments will be necessary to describe the effects in the mouse model of subsequent B cell depletion after colonization or vaccination events on memory and recall humoral responses to *S. pneumoniae*.

Despite the marked decreases in antibody levels in our mouse model, B cell depleted mice were still maintained significant levels of protection against subsequent *S. pneumoniae* pneumonia challenge compared to uncolonized mice. After challenge, lung CFU were comparable in the colonized depleted and undepleted groups showing no loss of colonization induced protection against lung infection. There was a significant increase in the incidence of sepsis in the depleted compared to non-depleted colonized group (31% vs 0), but 91% of the uncolonized mice developed sepsis showing that colonization with *S. pneumoniae* still leads to significant improvements in systemic immunity even after B cell depletion. Colonization-acquired adaptive immunity against lung infection in mice has previously been shown to be dependent on T cell mediated immunity in combination with antibody response (32). Hence the lack of effect of B cell depletion on lung CFU during pneumonia challenge probably represents maintenance of post-colonization T-cell dependent immune responses. Furthermore, we observed small increases in splenic T cells number in the depleted mice which may have improved Th1 and Th17 responses to colonization. As antibodies are the main mediator of immunity against *S. pneumoniae* reaching the blood (25, 28, 32), the maintenance of partial protection against septicemia despite the profound effect of B cell depletion on antibody responses to colonization is more surprising and harder to explain. Possibly, in this model of pneumonia causing septicemia even a low level of antibody is adequate to rapidly clear *S. pneumoniae* that reach the blood. Alternatively, the maintenance of T cell mediated immunity could provide an antibody-independent immunity mediated by Th17 cells and neutrophils recruitment (54, 55). In addition, activation of regulatory T cells (Tregs) has been reported in B cell depleted mice (56, 57),, and Tregs can reduce inflammation during pneumococcal pneumonia which is associated with decreased bacterial dissemination from the lungs to the blood and improved survival (58, 59). Additional experiments combining B cell and T cell depletion will be able to define whether persisting immunity to *S. pneumoniae* in previously B cell depleted mice was dependent on T cells.

The results obtained from this study indicate that B cell depletion therapy in humans could impair naturally acquired antibody responses to *S. pneumoniae*, with marked effects on the ability of colonization to induce anti-protein responses and reductions in natural IgM recognition of *S. pneumoniae*. Despite these major effects on humoral immunity, there was a limited effect on susceptibility to *S. pneumoniae* pneumonia. However, B cell depletion treatment in human disease is often repeated on multiple occasions and is also used in subjects who frequently have additional immune impairment of multiple aspects of the immune system due to other immunosuppressive treatments, or due to the disease itself. These individuals may also be more susceptible to lung infection due to comorbidities, structural lung damage or their age. Hence, the effects of B cell depletion in humans on antibody recognition of *S. pneumoniae* could be more serious than in the healthy mouse model and warrant detailed evaluation.

## Data Availability Statement

The original contributions presented in the study are included in the article/**Supplementary Material**. Further inquiries can be directed to the corresponding author.

## Ethics Statement

All studies utilising mice were performed in accordance with United Kingdom Home Office Animals Scientific Procedures Act (1986) under PPL licence P64714548, and were approved by the UCL Ethics Committee. Animals were culled at predetermined time points or at the point at which they showed moderate signs of disease in accordance with the Home Office Licence.

## Author Contributions

GE designed and performed all experiments and wrote the manuscript. ER-S contributed to the mouse experiments. GE, PF, RA, RN, and AJ designed and produced the protein array. DG designed the MSD experiments. GW and JB led the design and setup of the project, and contributed to the writing of the manuscript. All authors contributed to the article and approved the submitted version.

## Funding

GE and ER-S are supported by MRC grants MR/R001871/1 and R/N02687X/1, respectively. This work was undertaken at UCL and mainly funded by Novartis Institute for BioMedical Research, other funds were provided by the Department of Health’s NIHR Biomedical Research Centre’s funding scheme.

## Conflict of Interest

GW was employed by the company Novartis Institute for BioMedical Research.

The remaining authors declare that the research was conducted in the absence of any commercial or financial relationships that could be construed as a potential conflict of interest.
